# Implementation of a “Patient Blood Management” program in medium sized hospitals: Results of a survey among German hemotherapists

**DOI:** 10.1002/hsr2.924

**Published:** 2022-11-18

**Authors:** Thomas Frietsch, Gerhard Wittenberg, Audrey Horn, Andrea U. Steinbicker

**Affiliations:** ^1^ German Interdisciplinary Task Force for Clinical Hemotherapy (IAKH) Marburg Germany; ^2^ Anesthesiology and Critical Care Medicine BG Trauma Center Ludwigshafen am Rhein Germany; ^3^ Department of Anesthesiology Perioperative and Pain Medicine Stanford University Stanford California USA; ^4^ Department of Anesthesiology, Intensive Care and Pain Medicine, University Hospital Münster University of Muenster Münster Germany

**Keywords:** health care, health economy, Patient Blood Management, PBM implementation, preoperative anemia

## Abstract

**Background and aims:**

Germany uses more blood transfusions than the majority of other countries. The objective of this study was to detect the degree of Patient Blood Management (PBM) implementation within Germany and to identify obstacles to establishing PBM programs.

**Methods:**

An electronical questionnaire containing 21 questions and 4 topics was sent in 2018 to the members of the German interdisciplinary hemotherapy (IAKH) society in Germany. The degree of PBM (described as pre‐, intra‐, postoperative period) was established via questions within the topics “management of preoperative anemia” (PA) (*n* = 5), “preoperative management and transfusion preparation” (*n* = 3), PBM organization and structure (*n* = 5), coagulation management (*n* = 3), perioperative transfusion performance and habits (*n* = 3), best practices and problems (*n* = 2).

**Results:**

533 German hospitals with transfusion activity received the questionnaire with a 32.5% response rate to the survey. A dedicated PBM program had not been established in a quarter of all small and medium sized institutions. Red blood cell transfusion was the only therapeutic option in a third of institutions. Approximately half of the hospitals did not use knowledge of PA rates or transfusion needs of surgical procedures. Institutions failed to implement PBM because of a lack of profit, workload, personnel shortage, and administrative support.

**Conclusion:**

PBM was not present in at least a quarter of the hospitals interrogated. Factors for improvement were the relationship between health care disciplines and sectors, economic incentives, inclusion of relevant disciplines, and the structure of the blood industry. To improve BPM implementation, hospitals need support to implement top‐down PBM projects.

## 1 INTRODUCTION

A recent audit in Europe reported considerable variation in the degree of Patient Blood Management (PBM) implementation across 10 surveyed centers.^1^ In Germany, orthopedic and cardiothoracic disciplines perform hemotherapy by transfusing autologous blood components. Stimulated by international publications, the PBM concept was a constant focus in Germany's scientific meetings for more than 10 years. The concept was addressed to clinicians as a bottom up strategy, since they are the ones administrating blood products to patients. Later on, a joint project of PBM from a University Hospital and public media addressed all shareholders such as patients and health care officials through a TV report about practical blood use.^2^ However, the following question arose: to what degree has the concept been implemented after a decade? Recent reports demonstrated that initiating the process of implementing PBM as a three‐column program was very difficult^3^ all over Europe. In Germany specifically, PBM levels seemed to be underdeveloped compared to other countries in Europe. An analysis of a large German health insurance company identified a waste of 1 million units of packed red cells (PRC) per year.^4^ In round 1200 german hospitals, with little variation over the last decade, approximal 3 Mio PRC were needed. In this context, obstacles to implementing and maintaining all PBM elements are still unclear.

Unlike other countries, blood transfusion is an easy and inexpensive therapy in Germany. The acquisition cost for a unit of leukocyte‐depleted packed red blood cells range from 80 to 120€, which includes the cost of delivery, testing, and over‐night fees. Allogeneic red blood cell units are usually from voluntary donors at blood donation services located outside of hospitals. In contrast, PBM implementation needs additional time, energy and manpower. A clear return‐of‐investment margin for the hospital is less likely as the savings are slim. Bottom‐up‐implementation of PBM requires physicians to actively develop a convincing economical argument. The smaller the institution, the less likely personnel, time and financial resources are available. Thus, it can be difficult for small to medium size hospitals to successfully implement a higher level of PBM.

**Figure 1:**
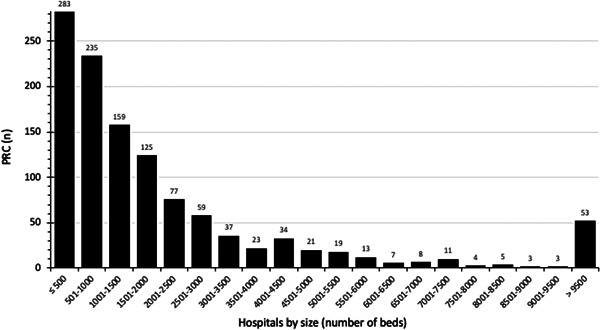
Blood Product use in Germany by number of hospitals. The number of packed red cells (PRC) is increasing from left to right. Columns represent the hospitals of the respective category (PRC use). Since among German hospitals (*n* = 1925 in 2018), smaller and medium size hospitals (less than 500 beds, *n* = 1584) are outnumbering bigger level one centers (500 and more beds, *n* = 280) by far,^5^ the conclusion is that small and medium size hospitals are contributing to the general transfusion activity in Germany considerably. Nationwide Trend data (not shown^6^) show that the number of transfusion activity reporting hospitals (approx. *n* = 1200) as well as the number of transfused PRCs (approx. 3 Mio) did not change much over the last decade. With permission from Thieme.^5^

However, in Germany, the majority of blood products are transfused in these smaller hospitals (Figure 1).^5^, ^6^ Among the 2525 blood using institutions and approximal 1200 hospitals that were reporting blood consumption in Germany in 2018 (median *n* = 1216 PRC in 2018), transfusion of PRC in hospitals with less than 500 beds is contributing to a major part to the nationwide consumption of blood products. Thus the practice habits in the smaller hospitals would be more relevant than those in the few university health care level I centers. In the latter, a high(er) degree of implementation can be assumed due to their initiative of PBM proclamation. However, neither the status of implementation nor the existence of potential barriers to a German PBM in smaller institutions were known.

Moreover, the German transfusion structure differs considerably from other European countries. In Germany, quality management and hemovigilance is devolved to “transfusion officers and commissioners” (see German transfusion law^7^). A dedicated transfusion law regulates a personal division of responsibility for the quality of hemotherapy, while practical hemotherapy is performed by clinicians of every discipline in adherence to medical guidelines.^8^, ^9^ The surveillance personnel of commissioners and officers does not necessarily include their involvement in diagnosis and treatment of anemia. Diagnosis of anemia is mostly performed by hematologists and oncologists; coagulation disorders require therapy from hemostaseologists. Anesthesiologist are in charge of perioperative management, including cell salvage.^10^ Because hemotherapy involve a multiplicity of responsibilities and players, implementation of a PBM concept might be more difficult in Germany than elsewhere. Perioperative aspects of PBM include various other clinical specialties. Therefore, the concept was spread for most of surgical and conservative clinical departments although dedicated PBM programs for nonsurgical specialties that is, oncologists were promoted be only a few societies in Germany.

To get reliable data about the PBM implementation throughout Germany, the “Interdisciplinary Working Group of Clinical Hemotherapy,” (IAKH, www.iakh.de, one of the promoting societies) sent a questionnaire to the members of the society to interrogate about the PBM implementation status of their affiliated hospitals. Since the IAKH supports the education of dedicated hemotherapists by evidence transfer to small and middle size institutions, their members would give a realistic status report about PBM implementation. In this article, we state the responses in detail and discuss the German‐wide implementation status.

## 2 MATERIALS AND METHODS

The survey questions were created by the research team, an advisory board from quality assurance, data management of IAKH, and the local medical association. Two experts in the field were asked to review all items of the questionnaire. An ethical committee was not necessary for this survey because there were no human subjects involved.

The survey was distributed through e‐mail as an online electronic questionnaire to eligible participants on April 22, 2018. The cutoff date for responses was September 24, 2018. We used an online survey tool from a web content management software (Surveil_CE, Contao vers. 2.11, 2012) to conduct the survey.

The study protocol was approved by the local ethical board. Participating physicians were anonymized via nontracible entries in an encrypted web‐based entry form. The IAKH hemotherapy society sent an electronically secured email to their members (*n* = 536) containing a description of the study and a survey link. The members of the IAKH are qualified transfusion commissioners and officers as well as the staff of quality assurance in hemotherapy. Of 536 members, *n* = 533 received the outgoing email. Three did not receive the email due to an invalid email‐address. Only one response per institution was accepted. The size of each hospital was determined by the number of surgical procedures per year.

The survey included 21 questions representing the pre‐, intra‐, postoperative period of PBM addressing the following topics: (1) Assessment of transfusion risk (an estimation of the average blood loss of a procedure in relationship to the erythrocyte mass of the patient), (2) existence of a PBM concept within the hospital, (3) Management of preoperative anemia (PA) (time frame and execution of diagnosis and treatment in the preoperative period), (4) Use of PBM elements (in elective and urgent situations, embedded in algorithms or SOPs), (5) Coagulation management, (6) Use of cellular blood products, (7) Quality management and structure, (8) Best practice.

Since the questionnaire was conducted in German, we translated the questions to English for purposes of the manuscript. The translated questions are listed in Table 1. The majority of questions (*n* = 19) have either five to seven predetermined potential answers to choose from, or an additional field listed as “other” for responders to type in their own responses. Two of the 19 questions were open ended. These questions were (1) to name one best hemotherapy practice example that is performed in their own hospital better than in other hospitals and (2) the difficulties in PBM introduction and implementation, management and the support required for the implementation.

**Table 1:** IAKH Questionnaire—PBM status in Germany

1	How many surgical procedures were performed in your institution last year/annually?
2	What is the rate of patients with preoperative anemia in your institution last year?
3	When do you have a hemoglobin level diagnosed preoperatively in your institution regularly (more than 50% of cases)?
4	How is the hemoglobin level in your institution measured before elective surgery?
5	What kind of anemia assessment is established in your in‐house laboratory?
6	Do you calculate erythrocyte mass and blood volume of your surgical patients?
7	How do you assess transfusion risk, blood loss and needed transfusion units?
8	Who is in charge of PBM in your institution? If not yet established what are the problems?
9	When is preoperative anemia treated preoperatively in your institution?
10	Who is/should be responsible for the treatment of preoperative anemia in your institution?
11	Which of the following PBM elements are used regularly (more than 2/3 of cases) in elective surgical cases?
12	Which of the following PBM elements are used regularly in urgent surgical cases?
13	Are the algorithms or SOPs for the use and dosing of iron, epo, etc.
14	Which instrument of coagulation management are used in your institution regularly (80% of cases)?
15	Is an in‐house‐specialist for hemostaseology available?
16	What is the rate of double unit orders of packed red cells (according to blood bank statistics)?
17	Is a blood ordering schedule for each surgical department and surgery type (OPS, procedure) established?
18	Are indications for the transfusion well documented (use 10 chart reviews from last year)?
19	How frequent the transfusion commission was having a meeting in your institution?
20	What is your best hemotherapy practice example, that in your institution is better than elsewhere? (open question)
21	Where are the difficulties in PBM introduction and management? For which elements or departments do you need what kind of support? (open question)

*Note*: Questions 1 to 19 were multiple choice questions with 2 to 7 responses possible, some with additional narrative options in the end. The last two questions were open narrative questions.

Abbreviations: PBM, Patient Blood Management; SOP, standard operating procedures.

The responses were transferred to Excel (vs. 16.16.4). Data analysis was descriptive. Since a detailed statistical analysis is not necessary to describe the survey's results, there were no significance level and methods to define.

## 3 RESULTS

**Figure 2:**
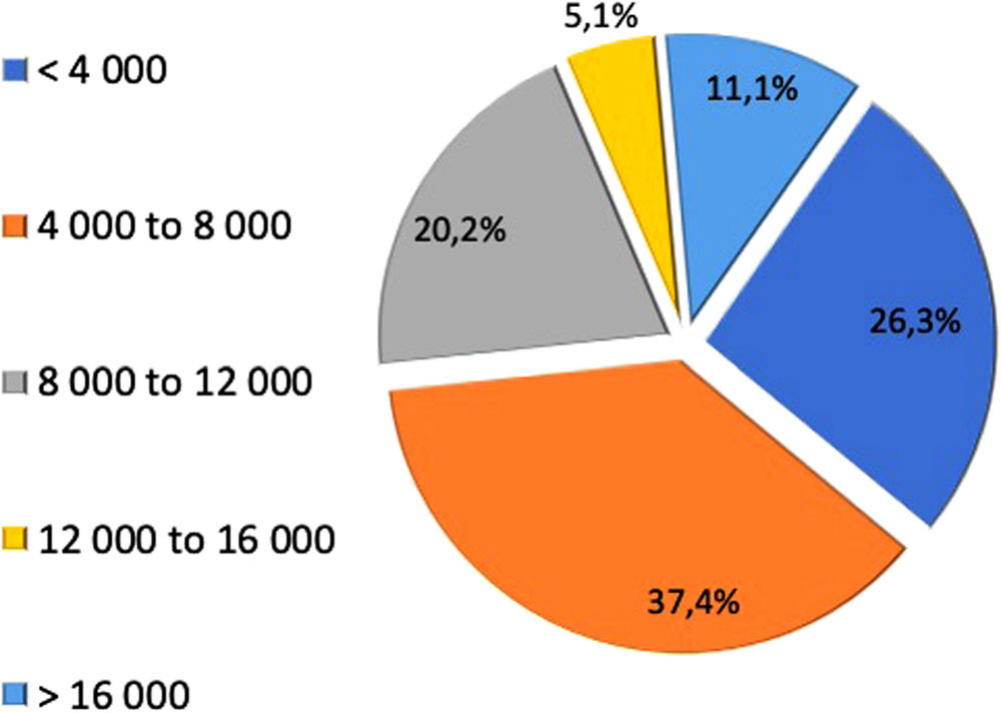
Size of health care institution by the number of surgical procedures per year. Contribution in the survey came from hospitals (responding institutions *n* = 99) with surgical activities in orthopedic, urologic, gynecologic, visceral, and vascular surgery. The cake graph represents hospitals with minor surgical activity in the lower half. The two categories of highest number of surgeries add up to 16.2% of responses. Survey participants originated from small or medium surgical centers.

The response rate to the survey was 32,5% (174 answers from 533 members of the IAKH hemotherapy society). About 64.4% of answers were received from small and medium size hospitals performing less than 8000 surgical interventions per year (Figure 2). Of the 64.4% of small and medium sized hospitals, 26.9% of institutions conducted less than 4000 surgical procedures per year and 37.5% between 4000 and 8000 procedures per year. Members of larger hospitals made 35.2% of the replies, of which 19.2% of answers came from institutions with 8000 to 12,000 surgical interventions per year, 4.8% between 12,000 and 16,000, and 11.2% more than 16,000 surgeries per year (Figure 2).

### 3.1 Transfusion risk assessment

From those institutions that had a SOP guided blood order schedule (BOS) as a preparation for major blood loss and perioperative transfusion, 58% were based on estimates of surgeons or experts. 32% of responders prepared their BOS according to the consumption of actual cases. About one in every 10 institutions (9%) used blood bank data (consumption and unused reservations per procedure) for BOS. In one institution, the BOS was not kept up to date and another responder adapted the statistical data derived estimated blood loss (EBL) based on their personal experience. In 4% of all responding institutions, the EBL of procedures was completely unknown.

Total blood volume or erythrocyte mass were calculated, if necessary, for special cases in 35% of institutions. In 64% of responding institutions, the relationship of EBL and patient's erythrocyte mass was not considered for preparation of red blood cell concentrates before surgery. One response given was that this method was declined by the transfusion commission of that institution.

### 3.2 PBM program and performance

The concept and program of PBM was introduced and maintained by the departments of anesthesiology (47%), transfusion medicine (11%), internal medicine (4%) or surgery (15%, containing all surgical disciplines). Surgery included abdominal surgery (5.6%), cardiac (2.3%), vascular (3.4%), gynecology/obstetrics (1.1%) and urology (2.3%). Twenty‐six percent of all institutions surveyed did not have an established PBM program. In 7.5% of the responding institutions, PBM was in the process of implementation, but had not yet been established at the time of survey. The major cause for failing to implement PBM was the lack of profit and reimbursement in relationship to an expected workload from the organization itself, a required change in management and complex interdisciplinary and intersectoral networking (25%). The other causes included the lack of available personnel resources (work overload and/or lack of staff in 15%), lack of interest and/or information of surgeons (12.5%), delay of elective surgery (12.5), resistance and lack of interest by hospital directory board (7.5%), rigid health care structures and lack of interest from nonhospital care physicians to participate (10%) in the program. Diagnosis and therapy of PA especially was seen as a hindrance to program implementation (12.5%) since hemoglobin levels were either taken too late for adequate anemia therapy, or were considered tolerable in most cases of “mild” anemia. Two trauma centers were unable to establish a PBM program due to their quick scheduling for urgent surgical procedures.

### 3.3 Preoperative Anemia (PA)

#### 3.3.1 Diagnosis

**Figure 3:**
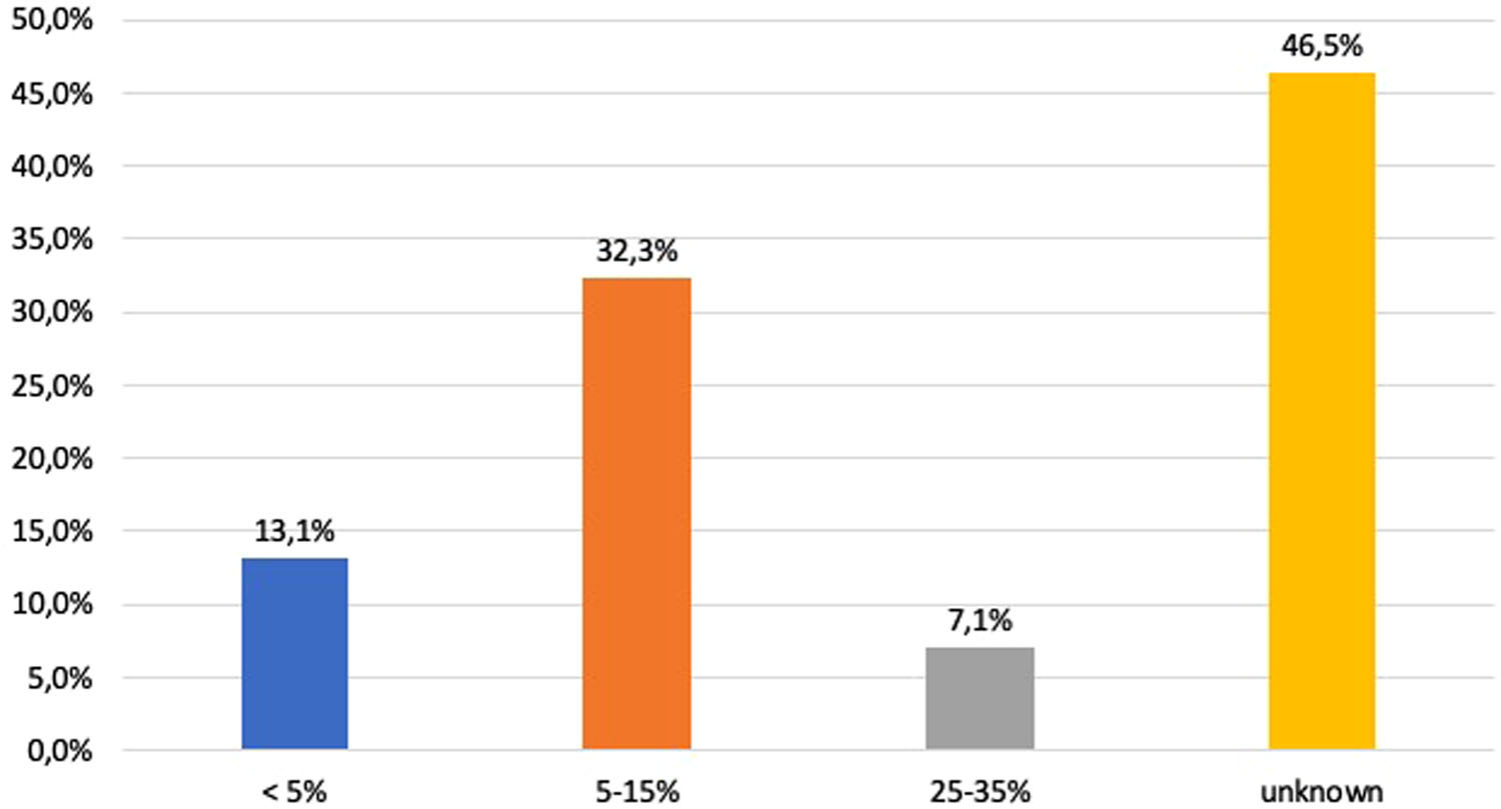
Ratio of preoperative anemic patients in elective surgery. Derived from responding institutions (*n* = 176), columns show the rate of anemia before elective surgery in participating hospitals. In almost half of institutions (46,5%), preoperative hemoglobin concentration is not known when surgery starts.

In nearly half of the responding institutions, the rate of PA was unknown (Figure 3). Hemoglobin levels before surgery were taken 1 day before surgery (57.1%), at the day of surgery (0.95%), 3 to 5 days before admission (23.8%) or up to 3 weeks before surgery (12.4%). In 77% of institutions, hemoglobin levels were unknown until the week before surgery. Measurements of Hb levels more than 7 days before surgery was only stated in 16% of treated patients. Only 20% of hospitals determined patient hemoglobin levels by their general practitioner or referring specialists. Nine percent of hospitals used noninvasive plethysmography as a screening tool for anemia. If a low hemoglobin concentration is detected, a proper laboratory work up for the blood based quantification, red cell morphometry, and causal diagnosis for iron deficiency or others is performed usually, according to an actual anemia guideline. More than half of institutions (56%) used automated cell counters as a method of in‐hospital hemoglobin measurement. Solely hemoglobin content was taken in 5% of institutions and further diagnostics eventually followed. An algorithm for anemia diagnosis was stated in only 1% of hospitals. Anemia assessments were analyzed by either ferritin levels alone (10.1%) or with additional transferrin saturation (16.2%). Although it was not a routine standard, reticulocyte hemoglobin was analyzed by 10.1% of hospitals. A preoperative calculation of the individual red cell mass and/or plasma volume was very rarely used (Figure 5).

#### 3.3.2 Therapy

**Figure 4:**
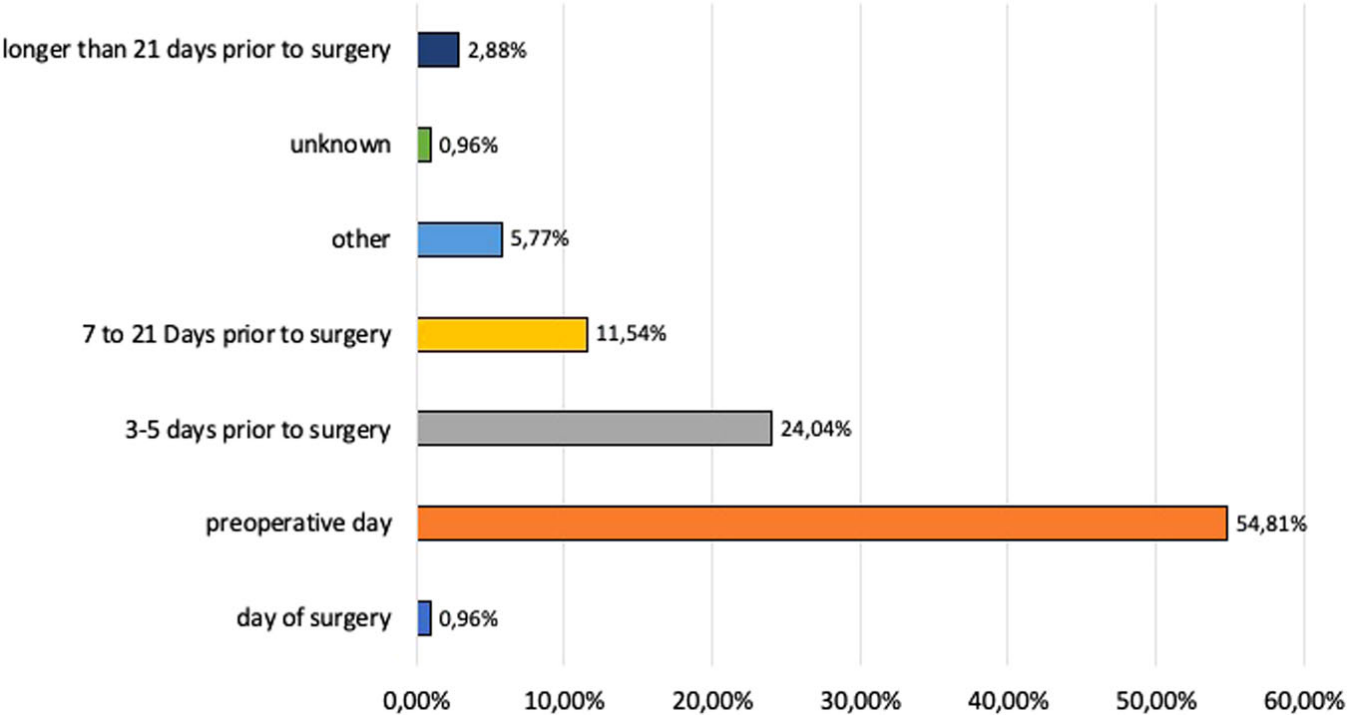
Time for preoperative measurement of hemoglobin content in elective surgery. The answers (*n* = 89) to question 3 (“In your institution, when do you measure hemoglobin content before elective surgery in more than 50% of procedures]?”) show that in Germany, the majority of patients were admitted the day before surgery and tested for hemoglobin contents.

In more than a third of the responding institutions (35%), management of PA was restricted to perioperative red blood cell transfusions. It was common in 24.7% of institutions to start adequate anemia therapy more than a week before surgery. Most institutions (47.1%) began therapy within the week before surgery (Figure 4). 23.5% of institutions started therapy either the day of surgery or 1 day before surgery. The responding physicians of this survey stated that responsibility for anemia diagnosis and therapy should be assumed by the following: general practitioner of the patient (43.4%), referring specialist (19.3%), cooperating ambulatory healthcare center (7.1%), dedicated PBM unit/autologous donation unit within the hospital (7.2%), admission unit (4.8%), patient logistics (3.6%), and/or the hematologist (1.2%) (Figure 6).

**Figure 5:**
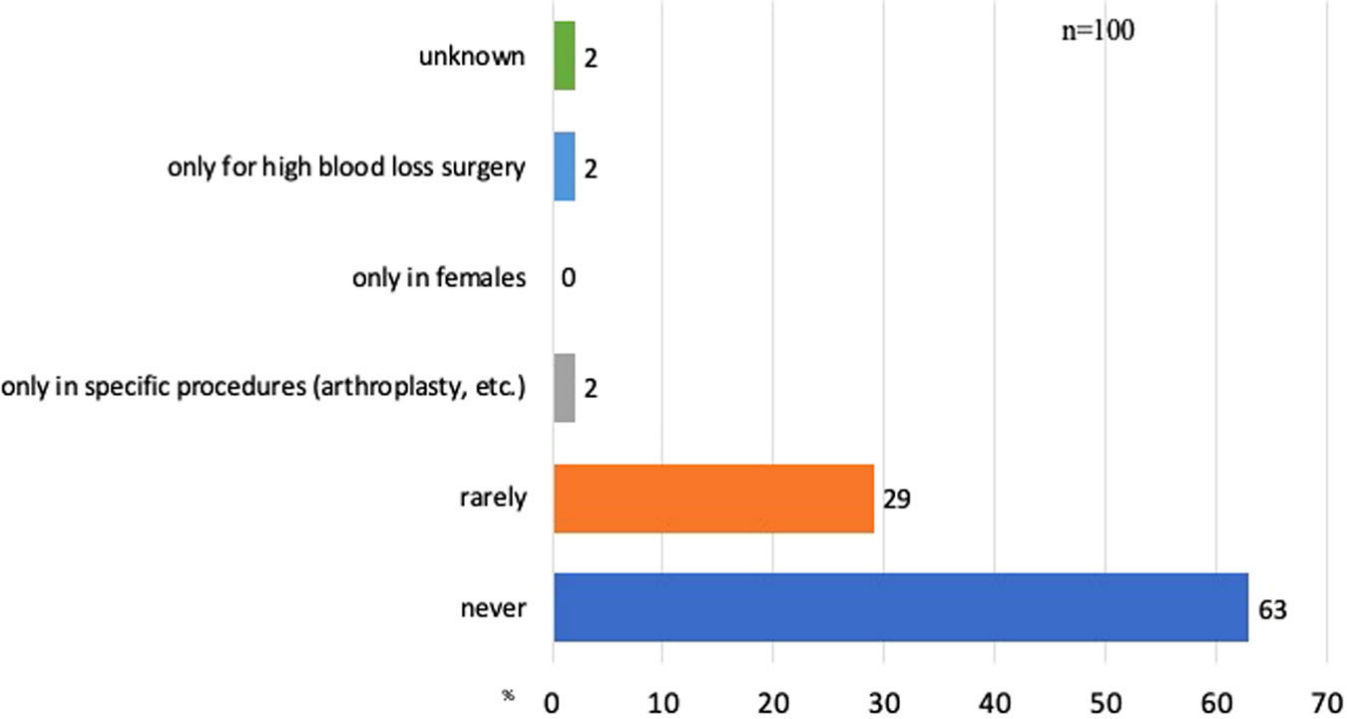
Preoperative calculation of individual red cell mass and blood volume. The answers (*n* = 182) to question 5 (“In your institution, the individual erythrocyte mass and blood volume of patients is calculated/estimated?”) show that only a small minority of hospitals individualize their preoperative assessment with relevance to transfusion requirements. Responding hospitals *n* = 100, numbers are absolute numbers or percentage.

**Figure 6:**
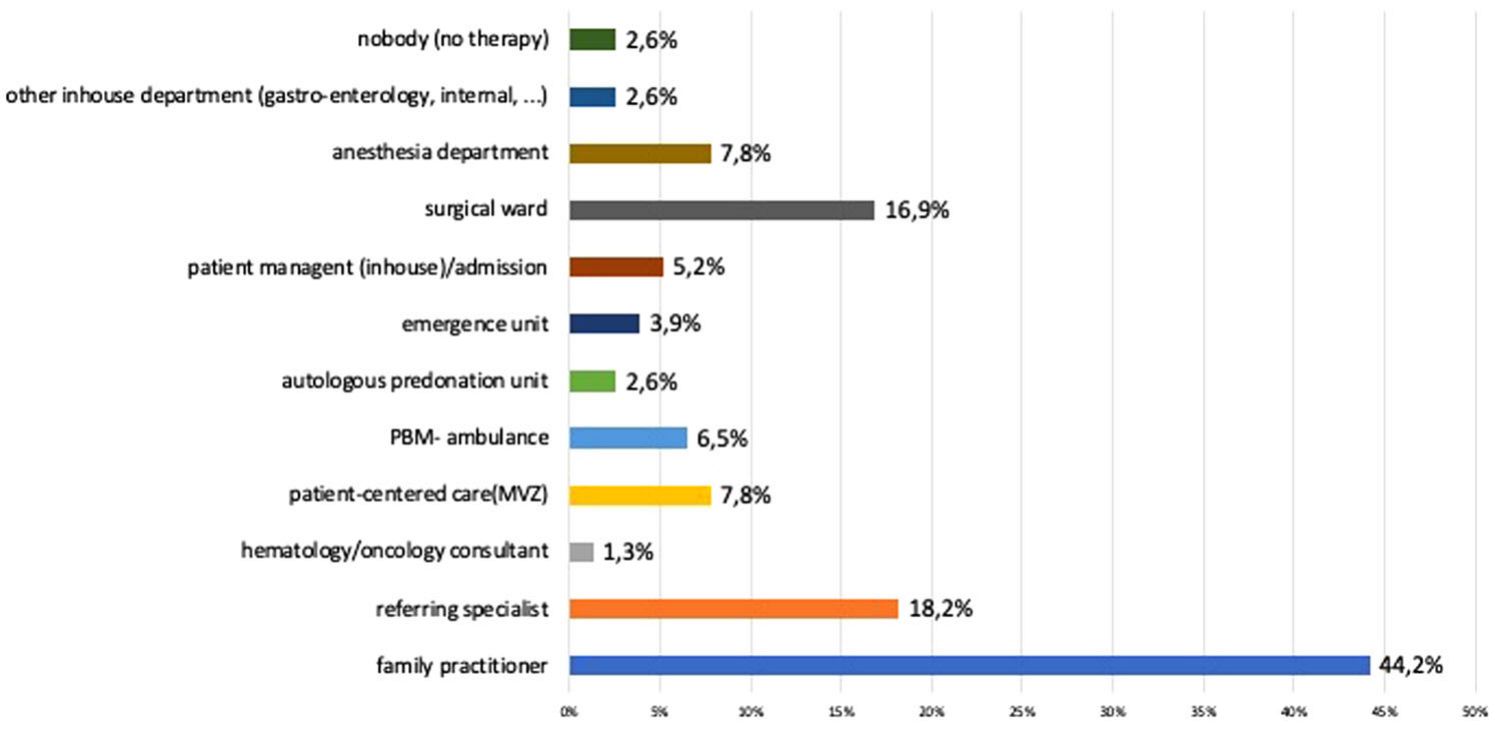
Responsibility for preoperative anemia therapy The answers (*n* = 190) to question 10 (“In your institution, who is in charge for therapy of preoperative anemia?”) show that there is no standard for anemia therapy in smaller hospitals. General practitioners are or should be by 44,2%. MVZ, German Medical Treatment Center; PBM, Patient Blood Management.

### 3.4 PBM elements used

**Figure 7:**
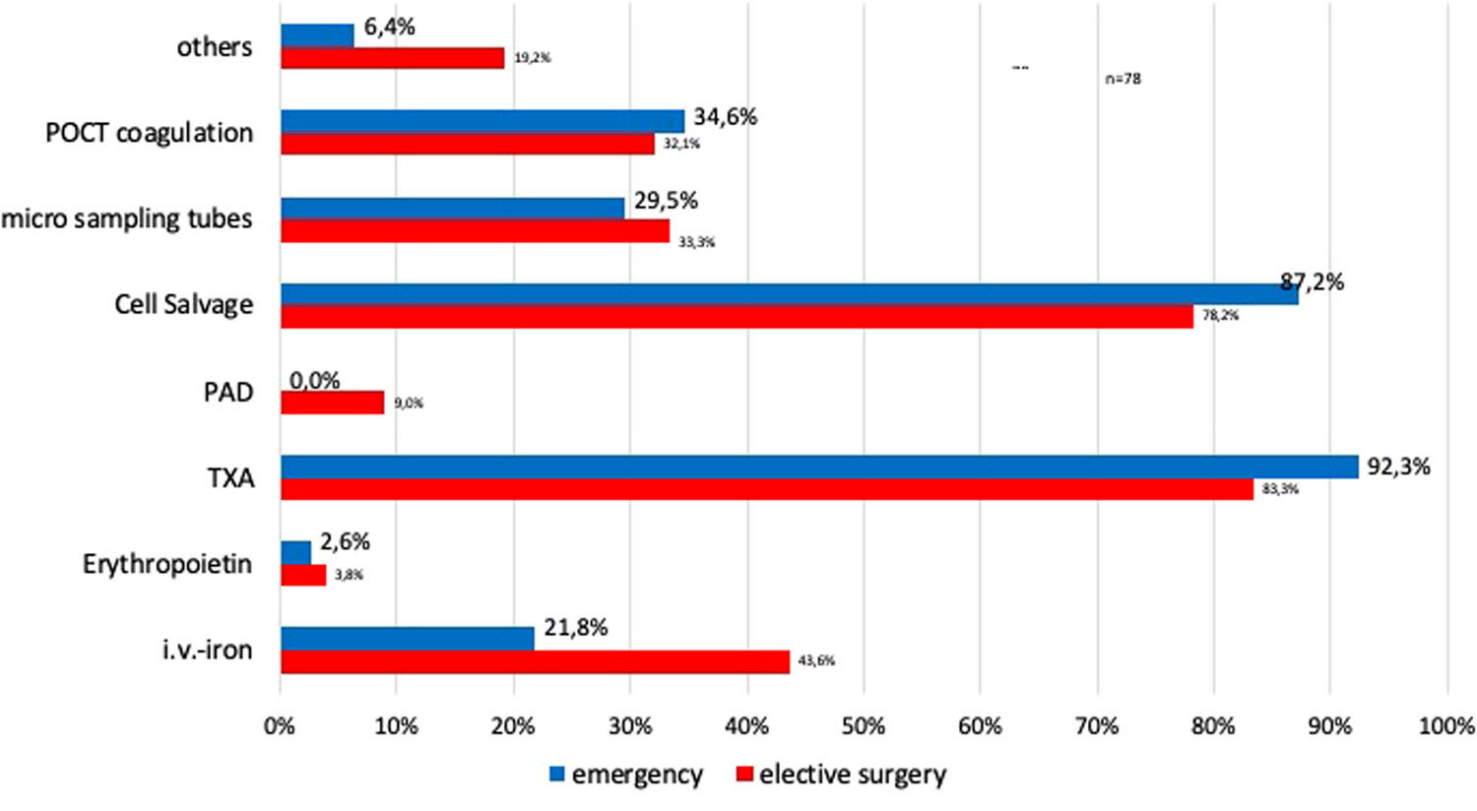
Established methods of PBM for elective and emergency surgery. The answers (*n* = 191 and *n* = 193) to questions 11 and 12 (“In your institution, what kind of PBM methods are established and commonly used in elective [Q11] or emergency [Q12]) cases (in more than two thirds of procedures?”). i.v., intravenous; PAD, preoperative autologous blood donation; PBM, Patient Blood Management; POCT, point of care testing; TX, tranexamic acid.

Among the seven given methods in the PBM concept, the two most common and established options in elective and emergency situations were tranexamic acid and autologous cell salvage (Figure 7). Standard operating procedures (SOPs) (including dosing schemes) plus algorithms were available for the following procedures (in %): Cell salvage (83.1%), tranexamic acid (57.8%), point‐of‐care diagnosis (POCT) coagulation management (29.6%), i.v. iron (28.2%), microsampling (9.9%), and erythropoietin (EPO, 7.0%).

### 3.5 Coagulation management

Consequent warming of patients was the most common technique in hospitals (86.6%). A standardized coagulation history and status were taken in a quarter of participating institutions (25.6%). An in‐house specialist for hemostaseology was available in 25.6%, but was only used in 15.9% of all responding institutions.

Standard diagnostic laboratory coagulation screening (coagulation markers PT or INR, aPTT) was used in 75.6% of hospitals, in conjunction with either fibrinogen levels or thrombin time/anti‐Xa in 36.6% and 20.1% of hospitals, respectively. Other common diagnostic parameters were acid–base‐status (69.5%) and calcium levels (68.3%). In‐house availability of diagnostic methods varied between institutions: Quantitative measurement of coagulation factors (9.8%), aggregometry (6.1%), platelet function testing (occlusion aggregometry PFA‐100) (10.1%), point‐of‐Care analyzers (thrombelastometry, thrombelastography, impedance aggregometry [20.1%]).

### 3.6 Transfusion practice

**Figure 8:**
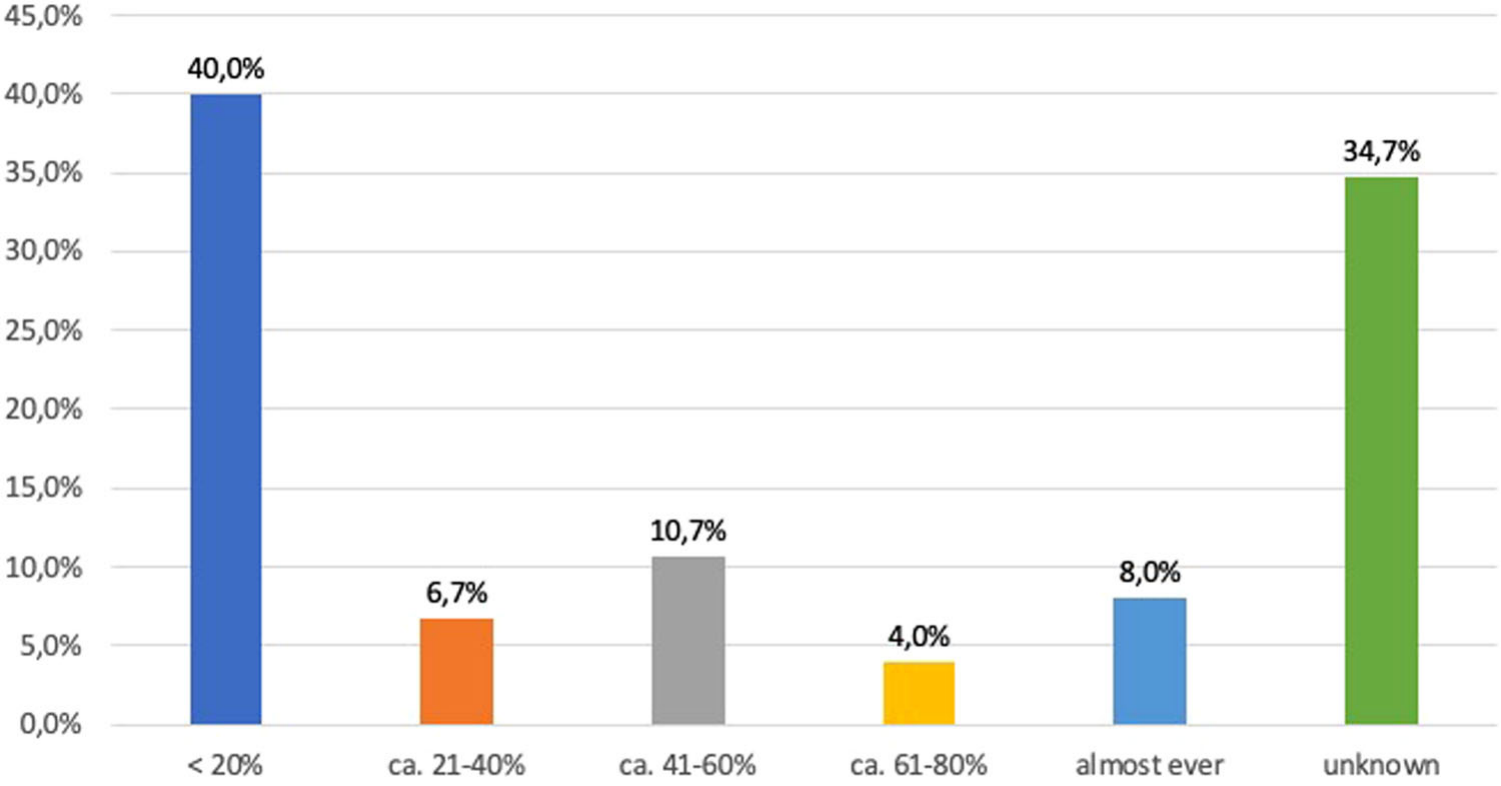
Order and administration of multiple units of packed red cells (PRC) in elective surgery. The answers (*n* = 75) to question 16 (“In your institution, what is the frequency of orders for two or more units of PRC for elective procedures [based on a statistical analysis of your blood bank]”) should reveal the practice of the transfusion of multiple units together. Based on the assumption that elective surgery does not produce uncontrolled bleeding in more than 20%, the answers can be interpreted that 40% of responding hospitals adhere to the single unit policy. In 8%, the administration of multiple units is standard of care (“almost ever”).

A common practice within institutions was to order double units of PRC as opposed to a single unit order: 41% of the responding institutions noted that double units were ordered in 20% to 80% of all orders (see Figure 8). One‐third of the responses (33.1%) indicated that data about transfusion statistics were not accessible and therefore unknown. In 17.1% of institutions, a perioperative BOS did not exist or an existing BOS was not used for the blood unit reservation. Documentation of transfusion indications varied by blood products; Although indications for red blood cell and platelet transfusions were well documented in most institutions (97.7% and 79.4%, respectively), the indication for plasma and fibrinogen (69.1% each) was not documented in nearly a third of institutions. The transfusion commission in most institutions have one meeting per year (56.1%). The other transfusion commissions met more than once a year (36.6%), every 2 years (4.9%), and less frequently than this in 1.2% of institutions.

### 3.7 Best practice in hemotherapy

Physicians (*n* = 40) replied to the open‐ended question about established best practice at their own institution. The most frequent answer was successfully introducing PBM aspects or single elements (*n* = 35). The frequency and type of methods used are listed in Table 2. In brief, 44 activities fitted into 13 categories. The use of restrictive transfusion triggers was included via educational information, transfusion lectures, guideline education, or cell salvage.

**Table 2:** Best practice methods (by frequency)

Method, PBM element	Number of counts (%)
Education (individually and/or collectively, statistical consumption analysis, SOPs)	8 (15.7)
Use of restrictive transfusion trigger, postoperative anemia tolerance	7 (13.7)
Transfusion medicine lecture, continuous medical education	5 (9.8)
Cell Salvage	5 (9.8)
Intraoperative heat conservation methods	4 (7.8)
Statistical analyses of blood products consumption	4 (7.8)
PBM Standard operating procedures or PBM algorithm	3 (5.8)
Tranexamic Acid	3 (5.8)
Coagulation management by point of care testing	3 (5.8)
Surgery postponement if preoperative anemia was diagnosed	3 (5.8)
Establishment of preoperative anemia diagnostics	2 (3.9)
Microsampling tubes for laboratory blood	2 (3.9)
Management of massive bleeding	2 (3.9)
Hemostaseology Counceling	1 (1.9)
Storage of ready‐to‐use lyophilized universal plasma	1 (1.9)
Blood ordering schedule for most of surgical procedures	1 (1.9)
Oral iron therapy before surgery	1 (1.9)
Single unit policy	1 (1.9)
Preoperative calculation of red cell volume	1 (1.9)
Personal engagement for PBM	1 (1.9)
Reliable look back procedure	1 (1.9)
Security of blood supply	1 (1.9)
Individualized hemotherapy	1 (1.9)

Abbreviations: i.v., intravenous; PBM, Patient Blood Management; POCT, point of care testing; SOP, standard operating procedures.

A mentioned complex aspect of PBM implementation was knowledge of institutional interdisciplinary structures and data assessment.

### 3.8 Difficulties in PBM implementation, management or the support required

The difficulties of implementing PBM are listed in Table 3. The biggest impediment to establishing PBM was the organization of diagnosis and therapy for PA. Three comments stated that their institution disregarded efforts for a higher degree of implementation in their institution since their restrictive transfusion policy as their first intervention was effective. After reducing the rate of blood consumption (between 40% and 70%), all other efforts were considered too expensive, unnecessary and disturbing. Other comments (*n* = 5) stated that PBM, diagnosis and treatment of PA confuses the established clinical pathway. The interdisciplinary communication and interaction were regarded as a challenge, especially difficult with a higher rate of out‐of‐house‐surgeons at smaller institutions. A considerably high number of responses listed the lack of interest and knowledge by other faculty members and other disciplines as a factor that prevent PBM implementation.

**Table 3:** Most important obstacles for the implementation of PBM

Difficulties to implement diagnostics for preoperative anemia	16
Difficulties to implement therapy for preoperative anemia	14
Missing interest/motivation of others in and out of own department	9
Missing support from superiors and administration	7
Organization work undone/missing	7
Tight scheduling (inclusive short period from anemia detection to date of surgery)	5
Missing cooperation between disciplines (i.e., from surgeons, nephrologists)	4
Missing cost compensation for anemia therapy	4
Problems with other intruments except preop. anemia (Cell Salvage, PBM Ambulance, POCT coagulation)	4
Software/IT	3
Missing assignment	3
Inability to change misconducts	3
Staffing shortage	2
Other inhouse structures (i.e., preoperative anesthesia assessment center)	2
Missing knowledge about PBM within the institution	2
Missing infrastructure (i.e., PBM Ambulance)/techniqual equipment (i.e., BGA, hemoglobin measurement)	2
Missing cooperation from external sector (i.e., referring physicians) in anemia management	1
Missing cooperation from internal departments (i.e., surgery) in anemia management	1
High ratio of emergency surgery	1
Missing SOP	1
Missing cost compensation for anemia diagnostic	1
Sufficient effect of other measures (i.e., Txa), no. need for PBM	1
Missing clinical pathway für preop. Anemia	1
Specific patient collective (wide catchment area)	1

Abbreviations: IT, intelligence technique; PBM, Patient Blood Management; POCT, point of care testing; SOP, standard operating procedures; Txa, tranexamic acid.

## 4 DISCUSSION/CONCLUSION

The major finding of this survey was that most of small to medium‐sized German hospitals had implemented at least one PBM method by 2018. However, multiple PBM efforts or measures from the pre‐, intra‐, postoperative period and qualifying for a full PBM program were only implemented in a small minority of institutions.

The distribution of hospital sizes in this questionnaire roughly represents the size of German health care: From 1925 hospitals in total,^11^, ^12^ greater hospital such as level one centers (more than 500 beds) represent less than a quarter of all institutions in Germany. The middle size and small hospitals, however, contribute to a majority in blood unit consumption. The IAKH survey reflects the PBM status in these institutions. Due to the promotion status of PBM in Germany and the distribution characteristics of conservative versus surgical PBM, mostly perioperative aspects of PBM are given (around 60% of the IAKH members are anesthesiologists). Of course, a retrospective, subjective questionnaire can only give a brief, descriptive insight into PBM implementation. Although we cannot draw full conclusions from this study, the data indicates that there is still a need to promote PBM implementation throughout Germany.

In some instances, PBM within Germany has become a highly certified and specialized in large University Centers (participants in the PBM network are 22 hospitals) whereas most of medium size and smaller hospitals were left behind. Despite the impressive increase in international PBM publication since its promotion in year 2008, the PBM as a concept was mentioned in German hemotherapy guidelines not before 2017 (the 2017 edition updated the 2010 version). This suggests that the penetrance of new practice improvements to smaller institutions is more delayed as opposed to academic teaching—a universal phenomenon.

A possible reason that Germany has a limited implementation may be due to their delayed initiative of PBM in comparison to other European Countries. The Netherlands, Australia, Italy, the UK, France, and Spain started earlier and installed PBM faster than Germany. Austria and Switzerland implemented at the same time as the German system.^13^ However, a recent survey about the implementation of PBM in seven European hospitals participating in the “PBM in Europe” working group of the “European Blood Alliance”—project uncovered significant knowledge gaps and a marginal level of PBM implementation.^3^ Participants of the survey by Manzini and colleagues differed, however, from our selection: Members of the PBM European Alliance working group emailed the web‐based form on a SurveyMonkey website to various clinicians in their hospital by an unknown selection mode. In contrast, the questionnaire in this German survey went to members of the IAKH society directly. As a conclusion, the Society's members' knowledge about the profits and quality improvements of a dedicated PBM establishment was assured. On the other hand, members of the IAKH society had expressed interest in hemotherapy and were therefore also biased and in some cases, were the ones in charge of PBM implementation.

Germany has the highest blood consumption per inhabitant (47.7 in 2015 in comparison to Austria 38.2; Norway 32.4; Australia 30.4; Japan 27.0 blood units per 1000 inhabitants).^4^, ^14^ One convincing explanation to the international variation is the “funding of blood support,” based either on governmental regulations or independent for‐profit businesses. In countries with national blood donation services, PBM concepts might be at a more progressive implementation level.

The way in which new practical guidelines are implemented might be a major contributing factor. As opposed to university centers, clinicians of smaller institutions are more focused to practical and economic aspects of their work. Guideline changes usually were faster implemented if there are promotors within the decision making level such as the hospital's board of directors (top‐down implementation). On the other hand, a bottom‐up initiative probably is more effective but slow. A conference initiated by a German Blood Donation Service about International PBM provoked some criticism.^15^ The implementation rather can be initiated by blood product producers with profit interest than by affected stakeholders such as patients and blood product users. The ideal mix between top‐down and bottom‐up obviously varies but has not been achieved for PBM in Germany yet.

Based on the results of this survey, the authors recommend various methods to incorporate PBM into hospitals. In brief, there are three, specific measures that combine bottom up and top down approaches. First, the knowledge gap between providers should be eliminated. This includes surgeons by informing them about outcome deferrals associated with allogeneic blood transfusions, as well as administrators about the associated costs for increases in hospital stays, complications, transfusion reactions, clerical errors and more. Furthermore, an obligatory benchmark tool should be introduced for both blood loss and procedure adjusted transfusion rates by the German physician association or a respected authority. In those institutions with high blood consumptions, a certification for PBM should be mandatory for the hospital with high blood loss and transfusion rate, based on external audits from an expert committee. Third, reimbursement for elective surgical procedures with untreated PA should be cut by insurances‐ for both‐coverage and treatment of complications and for the benefit of future patients.

To conclude, the current data show that in 2018, PBM implementation had reached a low grade. Programs to support the nation‐wide implementation should be strengthened, with a special focus on small and medium sized hospitals. An actual repetition of the questionnaire would give an impression about the current situation, which might have changed, especially due to the SARS‐CoV2 pandemics with shorter blood supply than in former times.

The re‐evaluation of implementation barriers would enable effective management change. Special attention and support should be given to profit and reimbursement, workload, personnel shortage, lack of administrative support and insufficient change management.

## AUTHOR CONTRIBUTIONS

**Thomas Frietsch**: Conceptualization; data curation; formal analysis; investigation; methodology; project administration; supervision; writing – original draft; writing – review & editing. **Gerhard Wittenberg**: Conceptualization; data curation; formal analysis; investigation; methodology; writing – original draft; writing – review & editing. **Audrey Horn**: Validation; writing – original draft; writing – review & editing. **Andrea U Steinbicker**: Data curation; formal analysis; project administration; writing – original draft; writing – review & editing.

## CONFLICT OF INTEREST

T. F. had limited consultant contracts with Janssen Cilag, Haemonetics, Vifor Pharmaceuticals and Pharmacosmos and received honoraria for scientific lectures from Janssen Cilag and Astra Zeneca, travel reimbursements from Janssen Cilag, Astra Zeneca, Braun, Behring CSL in the past. The remaining authors declare no conflict of interest.

## ETHICS STATEMENT

Ethical approval was not required or obtained, since neither patient's individual data nor personal involvement were requested. The contributing institutions and members of the IAKH rested unknown, because the response to the questionnaire was anonymous.

## TRANSPARENCY STATEMENT

The lead author Thomas Frietsch affirms that this manuscript is an honest, accurate, and transparent account of the study being reported; that no important aspects of the study have been omitted; and that any discrepancies from the study as planned (and, if relevant, registered) have been explained.

## Data Availability

The authors confirm that the data supporting the findings of this study are available on www.Iakh.de
